# Hemoglobin A1c and 10-year information processing speed in Japanese community dwellers

**DOI:** 10.1186/s12199-019-0778-8

**Published:** 2019-04-23

**Authors:** Rei Otsuka, Yukiko Nishita, Chikako Tange, Makiko Tomida, Fujiko Ando, Hiroshi Shimokata

**Affiliations:** 1 Section of NILS-LSA, National Center for Geriatrics and Gerontology, 7-430, Morioka-cho, Obu, Aichi 474-8511 Japan; 2 Faculty of Health and Medical Sciences, Aichi Shukutoku University, Katahira 2- 9, Nagakute, Aichi 480-1197 Japan; 3 Graduate School of Nutritional Sciences, Nagoya University of Arts and Sciences, Takenoyama 57, Iwasaki-cho, Nisshin, Aichi 470-0196 Japan

**Keywords:** Information processing speed, Hemoglobin A1c, Longitudinal study, Community dwellers, Japanese

## Abstract

**Background:**

Hyperglycemia is believed to be a risk factor for cognitive decline, but the longitudinal relationship between hyperglycemia and cognitive decline in the Japanese population is unclear. The present study aimed to clarify the association between blood glucose levels and information processing ability in middle-aged and older adults.

**Methods:**

The subjects were 866 men and 815 women aged 40–79 years not taking medication for diabetes who participated in the first study wave (1997–2000) and then participated at least once in the subsequent six study waves (2000–2012) of the National Institute for Longevity Sciences—Longitudinal Study of Aging, Japan. Hemoglobin A1c (HbA1c) levels were categorized into four groups (< 5.6, 5.6 to < 6.0, 6.0 to < 6.5, ≥ 6.5%), and a mixed-effects model was used to evaluate the effects of the HbA1c level (four groups) on repeated measures of information processing speed. The models also included baseline age, body mass index, ethanol intake, smoking status, educational level, family income, and history of stroke, hypertension, heart disease, and dyslipidemia as covariates.

**Results:**

Mean (standard deviation) HbA1c and follow-up time in participants were 5.2 (0.5) % and 10.0 (3.6) years, respectively. A linear mixed model showed that the main effect of the four HbA1c groups on information processing ability was not significant in either men or women, but the interaction of HbA1c and time with information processing speed in the higher HbA1c level groups (≥ 6.5% group in men, 6.0 to < 6.5% and ≥ 6.5% groups in women) was significant compared to the lower HbA1c level (< 5.6%) group (*P* < 0.05). When the slope of information processing speed by HbA1c level at baseline was examined, the slope of information processing speed in the higher HbA1c level (≥ 6.5%) group was higher than in the lower HbA1c level (< 5.6%) group, both in men (− 0.31/year) and in women (− 0.30/year), as well as in women with an HbA1c level of 6.0 to < 6.5% (− 0.40/year).

**Conclusions:**

Higher baseline HbA1c was associated with greater subsequent decline in information processing ability in Japanese community dwellers, even with the pre-clinical HbA1c level (6.0 to < 6.5%) in women. The results suggest that good glycemic control or prevention of hyperglycemia may contribute to maintaining information processing ability.

**Electronic supplementary material:**

The online version of this article (10.1186/s12199-019-0778-8) contains supplementary material, which is available to authorized users.

## Background

Diabetes is believed to be a risk factor for cognitive decline and dementia [1, 2]. The relationship between diabetes and cognitive function is thought to be a process mediated by factors such as arteriosclerosis, microvascular disease, glucotoxicity, and impaired insulin action [3].

Hemoglobin A1c (HbA1c) is generally well known and widely used as a marker reflecting the blood glucose level over the previous 1–2 months in health checkups. A persistent condition of high blood glucose levels might increase the risk of cognitive decline through the mediation of factors such as arteriosclerosis or glucotoxicity [3], and a cross-sectional study has reported a negative relationship between high HbA1c levels and cognitive function in diabetic patients [4]. However, the results of longitudinal studies do not agree with this relationship [2, 5].

In the Atherosclerosis Risk in Communities (ARIC) study, a 6-year follow-up study of diabetic and non-diabetic patients, diabetic patients had lower scores on the digit symbol substitution test (DSST), a test of information processing speed, but no significant relationship between HbA1c and cognitive function was found in non-diabetic patients [2]. A recent study conducted in the USA reported that a higher HbA1c was associated with poor executive function in persons with cognitive impairment, but not with performance in other cognitive domains [6]. Another English longitudinal study of aging (ELSA) reported a significant linear association between HbA1c and the increase in the rate of decline of global cognitive *z* scores [7]. A Finnish nationwide population-based study with 11-year follow-up reported that higher baseline HOMA-IR and fasting insulin levels predicted poorer verbal fluency performance and verbal fluency, but not word-list learning or word-list delayed recall scores [8].

In the Hisayama cohort study of Japanese community-dwelling people, a significant relationship was found between abnormal glucose tolerance evaluated by a 75-g oral glucose tolerance test and onset of dementia [9]. In the same cohort study, a significant relationship was found between the glycated albumin/HbA1c ratio and onset of Alzheimer’s disease, but HbA1c showed no significant relationship with onset of Alzheimer’s disease [10]. In the KOCOA Project, a 3-year follow-up study of Japanese oldest old (aged 80 years and older) persons, metabolic syndrome and four components including hyperglycemia were not associated with the decline in global and executive cognitive functions. However, high HbA1c (≥ 5.4%) was associated with a decline in memory function [11]. The relationship between HbA1c and cognitive function has not yet been sufficiently clarified in Japanese middle-aged and older persons. Clarifying the effects of the hyperglycemic condition on cognitive function is of great importance from the perspective of prevention of dementia in Japan.

The aim of the present study was to clarify the association between the HbA1c level and the changes in information processing ability in Japanese community-dwelling, middle-aged to older adults by means of a 10-year follow-up study.

## Methods

### Participants

Data for this survey were collected as part of the National Institute for Longevity Sciences—Longitudinal Study of Aging (NILS-LSA), a community-based study. In this project, the normal aging process has been assessed using detailed questionnaires and medical checkups, anthropometric measurements, physical fitness tests, and nutritional examinations over time. Details of the NILS-LSA have been reported elsewhere [12]. The initial survey of the NILS-LSA involved 2267 men and women aged between 40 and 79 years, including approximately 280 men and 280 women for each decade of age. Participants included were sex- and decade of age-stratified, randomly sampled individuals living in Obu-shi and Higashiura-cho, Aichi Prefecture, Japan. These subjects had been followed-up every 2 years from the first study wave (November 1997–April 2000) to the second study wave (April 2000–May 2002), third study wave (May 2002–May 2004), fourth study wave (June 2004–July 2006), fifth study wave (July 2006–July 2008), sixth study wave (July 2008–July 2010), and seventh study wave (July 2010–July 2012). When participants could not be followed up (e.g., moved to another area, dropped out for personal reasons, or died), new age decade- and sex-matched participants were randomly recruited from the second to seventh study waves. All study waves included approximately 1200 men and 1100 women. For this study, participants who had participated in both the first study wave (*n* = 2267; age range, 40–79 years) and at least one study wave from the second to seventh study waves (*n* = 1919) were selected, since variables could be followed up at least once after the first study wave. Individuals who were on medication for diabetes mellitus at baseline (*n* = 95), and who had any missing data at baseline required for the statistical analysis, including fasting HbA1c levels (*n* = 1), information processing speed (*n* = 5), and potential confounders at baseline (*n* = 137), including weight, height, alcohol intake, household annual income, education, and history of stroke, dyslipidemia, hypertension, and heart disease, were excluded. Thus, the data of a total of 1681 Japanese individuals (866 men, 815 women) aged between 40 and 79 years in the first study wave of the NILS-LSA were available for analysis (Additional file 1: Figure S1). To simplify the analysis, age at first participation was used in all analyses.

The NILS-LSA followed the principles of the Declaration of Helsinki and the Ethical Guidelines for Epidemiological Research in Japan. The study was approved by the Ethics Committee of the National Center for Geriatrics and Gerontology (No. 899–3). Written, informed consent was obtained from all participants.

### Blood sampling and HbA1c analysis

On enrollment in the survey, venous blood was collected in tubes containing ethylenediaminetetraacetic acid (EDTA, disodium salt, 50 mM) after fasting for at least 12 h. HbA1c levels were measured by latex aggregation immunoassay (SRL, Tokyo, Japan). The values for HbA1c were estimated as National Glycohemoglobin Standardization Program equivalent values calculated using the following formula: HbA1c (%) = 1.02 × HbA1c (Japan Diabetes Society) (%) + 0.25% [13].

### Information processing speed

The Wechsler Adult Intelligence Scale (WAIS) is one of the most used tools for assessing intelligence [14]. In all study waves, information processing speed was assessed using the DSST in JWAIS-R-SF [15]. The trained testers (clinical psychologists or psychology graduate students) administered the test to each participant one on one. Participants were asked to write down the symbol that corresponded to a given number (as many as they could in 90 s, possible range 0–93). This subtest measured processing speed and visual-motor coordination [16].

### Other measurements

Weight and height were measured in the fasting state (around 9–10 am) to the nearest 0.1 kg and 0.1 cm, respectively, with participants wearing light clothing and no shoes. Body mass index (BMI) was calculated as the body weight in kilograms divided by the square of the height in meters.

Education (≤ 11 or ≥ 12 years of school), household annual income (< ¥5,500,000 (almost 50,000 US$) or ≥ ¥5,500,000), current smoking status (yes/no), and history of illness were collected using self-reported questionnaires. Concerning illnesses, information about past and current diabetes, stroke, dyslipidemia, hypertension, and heart disease was collected in four categories: 1, none; 2, on medication (on medication); 3, previously medicated; and 4, not treated. These illnesses except for diabetes were then re-categorized into two categories (yes or no; yes included 2, on medication; 3, previously medicated; and 4, not treated and no included 1, none).

Alcohol intake was assessed using a 3-day dietary record and calculated according to the Standard Tables of Foods Composition in Japan 2010 [17, 18]. These measurements were assessed in the first study wave.

Follow-up time (years) was calculated by the length of time that had elapsed since the day each participant entered the first study wave.

### Statistical analyses

All statistical analyses were conducted using SAS version 9.3 software (SAS Institute, Cary, NC) and performed separately by sex. The mixed-effects model (Proc Mixed) was used to evaluate the effects of the HbA1c level on repeated measures of information processing speed. This method is a generalized form of linear regression analysis that allows for repeated measures of each participant while accounting for the considerable variation across participants in overall average information processing speed. In addition, general linear mixed models can handle missing data, so all variable data during follow-up could be used. The HbA1c level was categorized into four groups (< 5.6, 5.6 to < 6.0, 6.0 to < 6.5, ≥ 6.5%) by reference to the Evidence-based Practice Guideline for the Treatment for Diabetes in Japan 2016 [19, 20].

Analytical models included the fixed effects of baseline HbA1c level, follow-up time from baseline, and the interaction of HbA1c level and time, which showed the mean-level intercept and slope of information processing speed. Models also included baseline age, BMI (kg/m^2^), ethanol intake (ml/day), current smoking status (yes or no), educational level (≤ 9 or > 9 years), family income (< 5,500,000 or ≥ 5,500,000 yen/year), and history of stroke, hypertension, heart disease, and dyslipidemia (yes or no) as covariates.

In sub-analyses, multivariable-adjusted information processing speed (analysis of covariance) according to baseline HbA1c level was estimated in each study wave, adjusted for age, body mass index, ethanol intake, smoking status, educational level, family income, and history of stroke, hypertension, heart disease, and dyslipidemia.

All reported *P* values are two-sided. Values of *P* < 0.05 were considered significant.

## Results

Mean (standard deviation (SD), range) HbA1c, follow-up time, and number of study wave visits of participants were 5.2 (0.5, 3.6–9.4) %, 10.0 (3.6, 1.9–13.7) years, and 5.7 (1.8, 2–7), respectively.

The baseline characteristics of the participants according to the HbA1c levels are shown in Table 1. In both men and women, higher HbA1c was associated with higher age and lower information processing speed.

**Table 1:** Baseline characteristics of the participants according to HbA1c level

	Men (*n* = 866)					Women (*n* = 815)				
	HbA1c level				*p* ^a^	HbA1c level				*p* ^a^
	< 5.6%	5.6 to < 6.0%	6.0 to < 6.5%	≥ 6.5%		< 5.6%	5.6 to < 6.0%	6.0 to < 6.5%	≥ 6.5%	
	*n* = 549	*n* = 214	*n* = 67	*n* = 36		*n* = 571	*n* = 175	*n* = 44	*n* = 25	
Age (years), mean (SD)	57.2 (10.6)	59.8 (10.4)	60.3 (10.1)	61.4 (9.5)	< .01	55.6 (10.1)	59.9 (9.7)	65.9 (7.7)	61.9 (10.4)	< .01
Body mass index (kg/m^2^), mean (SD)	22.9 (2.6)	23.2 (2.7)	23.1 (3.1)	23.8 (3.2)	.20	22.4 (2.8)	23.6 (3.3)	23.7 (3.4)	27.4 (4.4)	< .01
HbA1c (%), mean (SD)	5.3 (0.2)	5.8 (0.1)	6.2 (0.1)	7.3 (0.8)	< .01	5.3 (0.2)	5.8 (0.1)	6.2 (0.1)	7.3 (0.9)	< .01
Information processing speed, mean (SD)	54.5 (14.9)	50.1 (13.7)	49.6 (12.8)	47.2 (13.2)	< .01	56.7 (15.5)	51.7 (14.8)	43.6 (12.6)	48.2 (12.2)	< .01
Ethanol intake (ml/day), mean (SD)	15.6 (17.6)	17.5 (21.2)	21.5 (21.6)	22.5 (23.2)	.02	3.2 (6.6)	2.0 (4.1)	4.0 (5.5)	1.5 (2.6)	.04
Current smoker, *n* (%)	172 (31.3)	103 (48.1)	26 (38.1)	16 (44.4)	< .01	42 (7.4)	12 (6.9)	5 (11.4)	0 (0.0)	.40
Education, ≤ 12 years, *n* (%)	362 (65.8)	157 (73.4)	51 (76.1)	26 (72.2)	.03	408 (71.5)	134 (76.6)	41 (93.2)	23 (92.0)	< .01
Family income, < 5,500,000 yen/year, *n* (%)	184 (33.5)	86 (40.2)	24 (35.8)	16 (44.4)	.24	221 (38.7)	87 (49.7)	27 (61.4)	10 (40.0)	< .01
Stroke, *n* (%)	17 (3.1)	10 (4.7)	0 (0.0)	1 (2.8)	.26	4 (0.7)	0 (0.0)	0 (0.0)	0 (0.0)	.70
Hypertension, *n* (%)	115 (21.0)	60 (28.0)	11 (16.4)	9 (25.0)	.11	110 (19.3)	48 (27.4)	14 (31.8)	8 (32.0)	.02
Heart disease, *n* (%)	62 (11.3)	27 (12.6)	11 (16.4)	5 (13.8)	.64	46 (8.1)	13 (7.4)	8 (18.2)	2 (8.0)	.14
Dyslipidemia, *n* (%)	65 (11.8)	37 (17.3)	8 (11.9)	5 (16.7)	.22	85 (14.9)	40 (22.9)	15 (34.1)	10 (40.0)	< .01

*HbA1c* hemoglobin A1c, *SD* standard deviation

^a^Chi-squared test or Fisher’s exact test (*n* < 5) for categorical variables and one-way analysis for variance for continuous variables

**Fig. 1:**
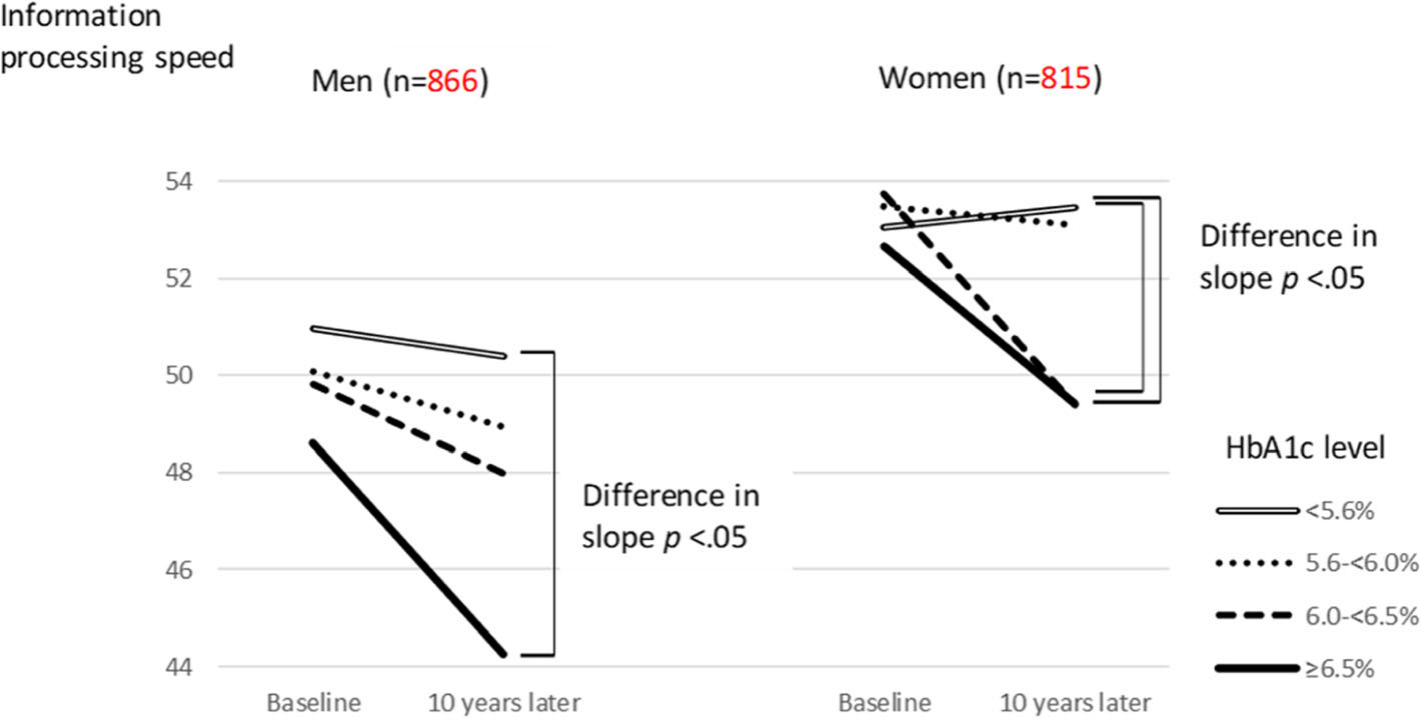
Model-predicted 10-year change in information processing speed according to baseline HbA1c level. Adjusted for age, body mass index, ethanol intake, smoking status, educational level, family income, and history of stroke, hypertension, heart disease, and dyslipidemia

Effects of variables in the mixed-effects model on information processing speed are shown in Table 2. In both men and women, the HbA1c level in each category was not significant. However, the interaction of HbA1c and time with information processing speed in the higher HbA1c level groups (≥ 6.5% group in men; 6.0 to < 6.5% and ≥ 6.5% groups in women) was significant compared to the lower HbA1c level group (< 5.6% group) (*P* < 0.05). When the slope of information processing speed according to HbA1c level at baseline was examined, the slope of information processing speed in the higher HbA1c level group (≥ 6.5% group) was higher than in the lower HbA1c level groups (< 5.6% group), both in men (− 0.31/year) and in women (− 0.30/year), as well as in women with an HbA1c level of 6.0 to < 6.5% (− 0.39/year) (Fig. 1).

**Table 2:** Effects of variables in the mixed-effects model^a^ on information processing speed

	Men (*n* = 866)				Women (*n* = 815)			
	*β*	(95%CI)	SE	*P*	*β*	(95%CI)	SE	*P*
HbA1c level								
<95%CI)5.6%	Ref				Ref			
5.6 to < 6.0%	− 0.90	(− 2.43, 0.63)	0.78	.25	0.41	(− 1.22, 2.04)	0.83	.87
6.0 to < 6.5%	− 1.15	(− 3.58, 1.28)	1.24	.35	0.69	(− 2.18, 3.66)	1.51	.95
≥ 6.5%	− 2.35	(− 5.58, 0.87)	1.64	.15	− 0.39	(− 4.32, 3.53)	2.00	.60
Time^b^	− 0.05	(− 0.10, 0.005)	0.03	.07	0.03	(− 0.02, 0.08)	0.03	.18
HbA1c level ×time^b^								
< 5.6%	Ref				Ref			
5.6 to < 6.0%	− 0.05	(− 0.15, 0.06)	0.05	.45	− 0.07	(− 0.17, 0.04)	0.06	.24
6.0 to < 6.5%	− 0.11	(− 0.28, 0.06)	0.09	.22	− 0.39	(− 0.60, − 0.19)	0.10	< .01
≥ 6.5%	− 0.31	(− 0.55, − 0.08)	0.12	<.01	− 0.30	(− 0.57, − 0.04)	0.14	.02

*HbA1c* hemoglobin A1c, *CI* confidence interval, *SE* standard error

^a^Adjusted for age, body mass index, ethanol intake, smoking status, educational level, family income, and history of stroke, hypertension, heart disease, and dyslipidemia

^b^Time (year) from baseline

In the sub-analyses (Additional file 1: Figure S2), multivariable-adjusted information processing speed according to baseline HbA1c level was examined in each study wave. There were no linear dose-response relationships between baseline HbA1c level and information processing speed in both sexes from baseline to third study waves. Significant linear associations (*P* < 0.05 and *P* for trend < 0.05) were seen in the fourth and sixth study waves in men and in the seventh study wave in both men and women.

## Discussion

These longitudinal data are the first to show that higher baseline HbA1c was associated with greater subsequent decline in information processing ability in Japanese community dwellers, even with the pre-clinical HbA1c level (6.0 to < 6.5%) in women.

Numerous cross-sectional studies reported that hyperglycemia or diabetes is related to cognitive impairment [2, 3], and the results are consistent. However, the results of longitudinal studies are not consistent [2, 5, 7, 8]. In the ARIC study with 6-year follow-up, there was a reduction in the DSST score (information processing speed) in diabetic patients, which was similar to the present study, while no significant relationship was found in the non-diabetic group from which self-reported diabetic patients were excluded [2]. The ARIC study divided non-diabetic patients into three groups (HbA1c < 5.7, 5.7 to < 6.5, ≥ 6.5%) on the basis of the 2010 guidelines of the American Diabetes Association to investigate the relationship with changes in the DSST over the 6-year follow-up (at two time points), but there was no significant relationship. They discussed the reasons as including that the subjects were mainly middle-aged (mean age 56 years) and the short follow-up. In addition, the recent long-term ARIC study (over 21 years) indicated that in subjects with diagnosed diabetes or HbA1c ≥ 6.5%, glucose peaks in addition to average glycemia may be important for prevention of dementia [20]. On the other hand, the Action to Control Cardiovascular Risk in Diabetes-Memory in Diabetes study found a dose-response relationship, with an increase in HbA1c associated with a 1.75-point drop in the DSST score in middle-aged to older subjects (mean age 63 years) with type 2 diabetes or other cardiovascular risk factors. However, this was a cross-sectional study. Furthermore, Bruce et al. carried out a 7-year follow-up of diabetic patients aged 70 years or over, and they reported that, while the number of years of diabetes duration and arteriosclerosis were risk factors for dementia, HbA1c, insulin, or hypertension were not independent risk factors [21].

On the other hand, Umegaki et al. reported a 6-year pooled analysis of type 2 diabetic patients in which reduction in cognitive function was significantly associated with low high-density lipoprotein values and high diastolic blood pressure values. While HbA1c showed no significant differences, they discussed the possibility that HbA1c might affect cognitive function [22]. In the present study, subjects on medication for diabetes at baseline were excluded. However, several subjects not on medication for diabetes had been diagnosed and were not on treatment for diabetes (men, *n* = 20 (2.3% in *n* = 866); women, *n* = 6 (0.7% in *n* = 815), data not shown). Although misclassification of the category of diabetes is unavoidable in this study, based on the slope of the information processing speed in the higher HbA1c level group (≥ 6.5% group) in both sexes and in the pre-clinical HbA1c level group (6.0 to < 6.5%) in women compared to the lower HbA1c level (< 5.6% group), the present study shows that long-term hyperglycemia in both sexes, even in the absence of diabetes in women, is related to future cognitive impairment.

The cascade linking hyperglycemia or diabetes to a reduction in cognitive function probably involves aging and genetic factors, as well as factors such as arteriosclerosis, microvascular disease, insufficient insulin action, oxidative stress, and glucotoxicity [3, 23]. In the present study, although the finding was not significant, men with lower HbA1c levels did not appear to have a lower prevalence of a history of stroke (3.1%, 4.7%, 0%, and 2.8% in the HbA1c < 5.6, 5.6 to < 6.0, 6.0 to < 6.5, and ≥ 6.5% groups, respectively, *P* = 0.26, Table 1), though the participants were not asked about the subtypes of stroke. The precise reason is unknown, but the study subjects were required to come to our center to have the study explained to them and to visit the examination center later; those with a history of more severe disease, such as stroke, were more likely to have been excluded (selection bias), even though the study subjects were randomly sampled individuals living in the local area. The present study was not able to examine these mechanisms in any detail, but it is likely that hyperglycemia impacts on reduction in cognitive function through the mediation of these in vivo reactions.

The main strengths of the present study were as follows. First, trends in information processing speed were assessed longitudinally (mean follow-up 10 years). Second, trends in these variables were analyzed by HbA1c level at baseline. In a prior study, we showed that information processing ability decreases gradually with age [16]. This is the first study to show that hyperglycemia has a negative impact on the gradual reduction in cognitive function among Japanese persons, even in the pre-clinical stage in women. In addition, the subjects were a random sample of general older adults from the community, stratified by age and sex. The present results may thus be more applicable to non-institutionalized, community-dwelling, middle-aged and older persons.

The present study had several limitations. First, hyperglycemia was assessed only by the initial level of HbA1c. HbA1c is a stable blood marker with relatively small daily variations, but the fact that HbA1c levels during the follow-up period were not considered is a limitation. The second is that the current/past histories and on medication/not on medication were defined by a self-reported questionnaire, which might have led to misclassification or underestimation of the prevalence of illness. In previous studies, community-dwelling older persons without any medical treatment were indifferent to health [24]; in fact, the subjects included participants with HbA1c up to nearly 10%, and although subjects were not on medication, subjects with an unquestionable possibility of having diabetes were included in the study. However, analysis of subjects excluding individuals with HbA1c ≥ 8% was conducted as a sub-study, and there was no major difference in the results. Third, there was no adjustment for confounding factors such as physical activity or dietary intake in the analyses. Not only these factors but also muscle mass or muscle strength has been found to be associated with diabetes mellitus [25], and these factors might have affected the results through motor or nutritional dysfunction. Fourth, it was thought that a generalized form of linear regression analysis, but not non-linear analyses (for example, a model including quadratic terms), would be better to assess longitudinal changes in these factors.

Another limitation that should be considered is based on age differences according to baseline HbA1c level. There was a clear sex difference in that only women, not men, with a pre-clinical HbA1c level (6.0 to < 6.5%) showed decreased information processing speed over 10 years. However, the mean age of women with an HbA1c level of 6.0 to < 6.5% was higher than that of women in other categories (Table 1). One of the reasons why a significant association was seen only in women may have been the age difference at baseline. However, age and multivariable-adjusted information processing speed according to baseline HbA1c level were not significant in both sexes from baseline to the third study wave. Therefore, baseline age could not be a strong contributor to the causal association. In addition, significant linear associations were seen in the sixth study wave (almost 10 years after baseline) in men and the seventh study wave (almost 12 years after baseline) in both men and women. This might mean that persistent long-term high blood glucose levels increase the risk of cognitive decline.

## Conclusions

The present longitudinal study showed that a higher baseline HbA1c was associated with a greater subsequent decline in information processing ability among community dwellers, even with the pre-clinical HbA1c level (6.0 to < 6.5%) in women. The results suggest that good glycemic control or prevention of hyperglycemia may contribute to maintaining information processing ability in middle-aged and aged Japanese community dwellers. Further studies are needed to clarify the causal association between HbA1c and cognitive decline, especially in the Japanese population.

## Additional file

Additional file 1: **Figure S1.** Subjects included in this study. **Figure S2.** Multivariable-adjusted^a^ information processing speed (analysis of covariance) according to baseline HbA1c level for each study wave. ^a^Adjusted for age, body mass index, ethanol intake, smoking status, educational level, family income, and history of stroke, hypertension, heart disease, and dyslipidemia. (DOCX 136 kb)

## Abbreviations

BMI: Body mass index
CI: Confidence interval
DSST: Digit symbol substitution test
HbA1c: Hemoglobin A1c
NILS-LSA: National Institute for Longevity Sciences-Longitudinal Study of Aging

## References

[1] Cukierman T, Gerstein HC, Williamson JD (2005). Cognitive decline and dementia in diabetes--systematic overview of prospective observational studies. Diabetologia..

[2] Christman AL, Matsushita K, Gottesman RF, Mosley T, Alonso A, Coresh J (2011). Glycated haemoglobin and cognitive decline: the atherosclerosis risk in communities (ARIC) study. Diabetologia..

[3] Biessels GJ, Staekenborg S, Brunner E, Brayne C, Scheltens P (2006). Risk of dementia in diabetes mellitus: a systematic review. Lancet Neurol.

[4] Cukierman-Yaffe T, Gerstein HC, Williamson JD, Lazar RM, Lovato L, Miller ME (2009). Relationship between baseline glycemic control and cognitive function in individuals with type 2 diabetes and other cardiovascular risk factors: the action to control cardiovascular risk in diabetes-memory in diabetes (ACCORD-MIND) trial. Diabetes Care.

[5] Gao L, Matthews FE, Sargeant LA, Brayne C, MRC CFAS (2008). An investigation of the population impact of variation in HbA1c levels in older people in England and Wales: from a population based multi-centre longitudinal study. BMC Public Health.

[6] Pappas C, Small BJ, Andel R, Laczó J, Parizkova M, Ondrej L (2019). Blood glucose levels may exacerbate executive function deficits in older adults with cognitive impairment. J Alzheimers Dis.

[7] Zheng F, Yan L, Yang Z, Zhong B, Xie W (2018). HbA1c, diabetes and cognitive decline: the English longitudinal study of ageing. Diabetologia..

[8] Ekblad LL, Rinne JO, Puukka P, Laine H, Ahtiluoto S, Sulkava R (2017). Insulin resistance predicts cognitive decline: an 11-year follow-up of a nationally representative adult population sample. Diabetes Care.

[9] Ohara T, Doi Y, Ninomiya T, Hirakawa Y, Hata J, Iwaki T (2011). Glucose tolerance status and risk of dementia in the community: the Hisayama study. Neurology..

[10] Mukai N, Ohara T, Hata J, Hirakawa Y, Yoshida D, Kishimoto H (2017). Alternative measures of hyperglycemia and risk of Alzheimer's disease in the community: the Hisayama study. J Clin Endocrinol Metabolism.

[11] Katsumata Y, Todoriki H, Higashiuesato Y, Yasura S, Willcox DC, Ohya Y (2012). Metabolic syndrome and cognitive decline among the oldest old in Okinawa: in search of a mechanism. The KOCOA project. J Gerontol A Biol Sci Med Sci.

[12] Shimokata H, Ando F, Niino N (2000). A new comprehensive study on aging--the National Institute for longevity sciences, longitudinal study of aging (NILS-LSA). J Epidemiol.

[13] Kashiwagi A, Kasuga M, Araki E, Oka Y, Hanafusa T, Ito H (2012). International clinical harmonization of glycated hemoglobin in Japan: from Japan diabetes society to National Glycohemoglobin Standardization Program values. J Diabetes Investig.

[14] Wechsler, D. The measurement of adult intelligence, 3rd ed. Baltimore, OH: The Williams & Wilkins Company; 1944.

[15] Kobayashi S, Fujita K, Maekawa H (1993). Japanese Wechsler adult intelligence scale-revised short forms.

[16] Nishita Y, Tange C, Tomida M, Ando F, Shimokata H (2013). Does high educational level protect against intellectual decline in older adults? : a 10-year longitudinal study. Jpn Psychol Res.

[17] Imai T, Sakai S, Mori K, Ando F, Niino N, Shimokata H (2000). Nutritional assessments of 3-day dietary records in National Institute for longevity sciences--longitudinal study of aging (NILS-LSA). J Epidemiol..

[18] Policy Division, Science and technology policy bureau (2013). Standard tables of food composition in Japan 2010.

[19] The Japan Diabetes Society (2016). Practice guideline for the treatment for diabetes in Japan 2016.

[20] Tabák AG, Herder C, Rathmann W, Brunner EJ, Kivimäki M (2012). Prediabetes: a high-risk state for diabetes development. Lancet..

[21] Bruce DG, Davis WA, Casey GP, Starkstein SE, Clarnette RM, Foster JK (2008). Predictors of cognitive impairment and dementia in older people with diabetes. Diabetologia..

[22] Umegaki H, Iimuro S, Shinozaki T, Araki A, Sakurai T, Iijima K (2012). Risk factors associated with cognitive decline in the elderly with type 2 diabetes: pooled logistic analysis of a 6-year observation in the Japanese elderly diabetes intervention trial. Geriatr Gerontol Int.

[23] Ninomiya T (2014). Diabetes mellitus and dementia. Curr Diab Rep.

[24] Tomioka K, Kurumatani N, Hosoi H (2017). Cross-sectional association between medical expenses and intellectual activity in community-dwelling older adults. Environ Health Prev Med.

[25] Nomura T, Kawae T, Kataoka H, Ikeda Y (2018). Assessment of lower extremity muscle mass, muscle strength, and exercise therapy in elderly patients with diabetes mellitus. Environ Health Prev Med.

