# Supplementary material for: Hemoglobin A1c and 10-year information processing speed in Japanese community dwellers
Source: Environ Health Prev Med. 2019 Apr 23;24:24. doi: 10.1186/s12199-019-0778-8 (PMC6480813; doi:10.1186/s12199-019-0778-8)
Supplement: Supplementary file 1 — Figure S1. Subjects included in this study. Figure S2. Multivariable-adjusteda information processing speed (analysis of covariance) according to baseline HbA1c level for each study wave. aAdjusted for age, body mass index, ethanol intake, smoking status, educational level, family income, and history of stroke, hypertension, heart disease, and dyslipidemia. (DOCX 136 kb) [file 12199_2019_778_MOESM1_ESM.docx]

**Fig. S1.**

**Fig. S2.**
